# 926. Shorter. Safer. Better. Pledge Campaign to Increase Uptake of Shortest Effective Durations of Antimicrobial Therapy

**DOI:** 10.1093/ofid/ofac492.771

**Published:** 2022-12-15

**Authors:** Danielle Doughman, Ashley H Marx, Nikolaos Mavrogiorgos

**Affiliations:** University of North Carolina Medical Center, Chapel Hill, North Carolina; University of North Carolina Medical Center, Chapel Hill, North Carolina; University of North Carolina Medical Center, Chapel Hill, North Carolina

## Abstract

**Background:**

Patients often receive longer-than-needed antimicrobial treatment for common infections, when a shorter duration would be equally effective. To promote use of evidence-based durations, the University of North Carolina Medical Center’s (MC) Carolina Antimicrobial Stewardship Program (CASP) developed best practices (BPs) in 2020 to inform prescribing. CASP conducted a signature pledge campaign to encourage nurses, pharmacists, prescribers, and allies to know, use, and share BPs.

**Methods:**

CASP developed a pledge form on a password-protected MC intranet site. Participants selected way(s) to “action” their pledge from a pre-filled list. CASP delivered coordinated messages to nurses, pharmacists, prescribers, and allied groups using multiple platforms: tiered safety huddles, leadership meetings, MC news, email, social media, and the CASP website over a 35-day campaign period that culminated with Antibiotics Awareness Week.

Measures of campaign effectiveness included number of pledgers, intranet views of BPs, reported awareness and use of BPs by pledgers at the time of pledge and in a 90-days post-campaign electronic survey, and pledge actions fulfilled.

Survey responses were cross-tabulated and analyzed using descriptive statistics.

**Results:**

238 MC staff signed the pledge; nurses comprised 21%, pharmacists 24%, prescribers 48%, and allies 7%. At time of pledge, 39% reported prior awareness of BPs (Figure 1); 39% reported prior use of BPs. 99% selected at least one action at time of pledge. 116 (49%) responded to the 90-day post campaign survey. 84% reported taking at least one action. The most common action reported among nurses was patient education (36%); among pharmacists, identifying too-long durations for query (78%); among prescribers, BP use and patient education were tied at 60%. The BPs page was viewed 323 times during campaign, compared to 153 total pre-campaign views. Prescribers’ BP use increased 66% from time of pledge.

90 days post-pledge, all groups except allies reported increased use of BPs since pledging (Figure 2).

Awareness of CASP Best Practices for Durations of Therapy resource among pledgers at time of signature.

**Figure:**
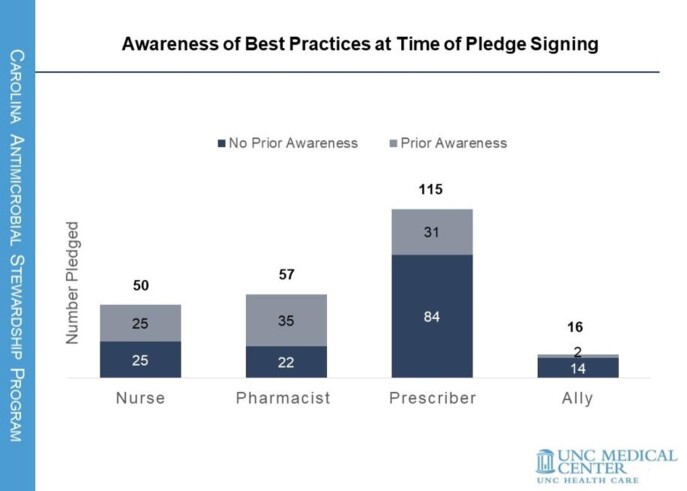

Reported use of CASP Best Practices for Duration of Therapy resource at the time of pledge and at time of survey, 90-days post campaign.

**Figure:**
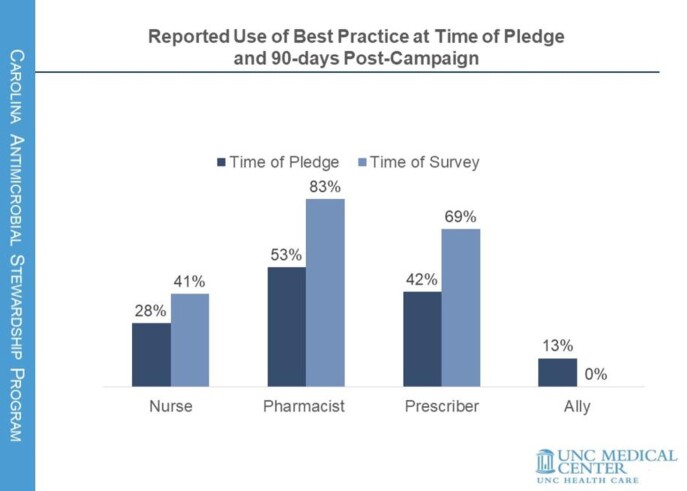

**Conclusion:**

A pledge campaign to increase awareness and uptake of shortest effective durations was effective except among allies. Further analysis is needed to determine if durations for common infections are similar to the BP ranges post-campaign.

**Disclosures:**

**All Authors**: No reported disclosures.

